# Microstructure and Friction–Wear Properties of 1Cr13 Coating on SAE 1045 Prepared by Arc Cladding

**DOI:** 10.3390/ma19061112

**Published:** 2026-03-13

**Authors:** Mengen Chen, Jufang Chen, Yu Zhu, Xiaoping Li

**Affiliations:** 1 School of Mechanical Engineering, Jiangsu University of Technology, Changzhou 213001, China; m18552907652@163.com (M.C.); lxp118@jsut.edu.cn (X.L.); 2 Shanghai Tianan Bearing Co., Ltd., Shanghai 201108, China; zhuyu361@126.com

**Keywords:** SAE 1045 surface, arc cladding, 1Cr13 coating, microstructure, friction and wear properties

## Abstract

**Highlights:**

**What are the main findings?**

- A 1Cr13 cladding layer was obtained on an SAE 1045 substrate through the arc cladding process.
- The average hardness of the coating reached 551.94 HV0.2, 2.26× of the substrate.
- The average friction coefficient of 1Cr13 (0.502~0.511) is lower and more stable than that of SAE 1045 (0.532~0.548) across all loads.

**What are the implications of the main findings?**

- Arc cladding leads to an increase in surface hardness and wear resistance.
- It provides a basis for iron-based wear-resistant coatings in engineering.
- It offers a low-cost method for strengthening medium-carbon steel parts.

**Abstract:**

To address the practical requirements for in situ equipment restoration, this study investigates a portable and cost-effective approach for the localized repair of SAE 10SAE 1045 components using a 1Cr13 martensitic stainless steel coating prepared via an arc-based additive manufacturing (WAAM) process. The microstructural evolution and tribological response of the layers were analyzed, with a focus on the effects of discrete thermal cycling and controlled solidification inherent to portable arc equipment. The WAAM process produced a refined martensitic matrix with a microhardness of 551.94 HV0.2, which is 2.26 times that of the substrate. Under dry sliding conditions, the 1Cr13 coating exhibited a lower friction coefficient and a reduced wear volume compared to the untreated SAE 1045, primarily through the mitigation of severe plastic deformation. This additive route provides a millimeter-scale reinforcement layer with metallurgical integrity suitable for heavy-duty service, aiming to offer a practical reference for the low-cost, on-site restoration of industrial components.

## 1. Introduction

SAE 1045 is a versatile medium-carbon steel extensively employed in the manufacturing of critical structural components, such as gears and shafts, due to its favorable balance of strength, ductility, and cost-effectiveness. However, its industrial application is often hindered by its limited hardenability and modest wear resistance under severe service conditions. Traditional heat treatments frequently struggle to provide sufficient functional depth for heavily worn parts. Consequently, utilizing portable arc-based additive manufacturing (WAAM) to repair and functionalize the surface of SAE 10SAE 1045 has become a pragmatic solution for extending the service life of equipment in field environments [1,2].

However, these components often operate under complex service environments and must endure prolonged cyclic loads. Their surfaces are highly susceptible to progressive damage caused by mechanical wear or electrochemical corrosion, which results in performance degradation, dimensional inaccuracies, and eventual structural failure. To prolong the service life of these components, the development of wear- and corrosion-resistant coatings on the surface of SAE 1045 has emerged as a significant research priority [2,3].

Wire Arc Additive Manufacturing (WAAM) represents an advanced metal additive manufacturing technology, colloquially known as 3D printing. Its fundamental principle relies on using an electric arc—such as Tungsten Inert Gas (TIG) or Metal Inert Gas (MIG)—as a high-energy-density heat source. During the fabrication process, a continuously fed metal wire is rapidly melted by the arc. Under precise computer control, the molten pool follows a predefined trajectory and solidifies, enabling the layer-by-layer construction of metal structural components characterized by macroscopic scales, highly dense microstructures, and excellent mechanical properties [3]. Compared with alternative arc-based techniques such as MIG and MAG, TIG (Tungsten Inert Gas) arc cladding offers distinct advantages for high-precision restoration, including a more concentrated heat source, lower dilution rates, and superior arc stability. While processes like FCAW provide higher deposition rates, TIG enables precise management of the discrete thermal cycling, which is critical for controlling the microstructural evolution and preventing cracking in 1Cr13 martensitic stainless steel. Furthermore, its simplified equipment and high wire utilization make TIG an ideal candidate for the on-site remanufacturing of industrial components with complex dimensions, where both portability and coating integrity are paramount. Arc-based additive manufacturing can be categorized into similar and dissimilar material deposition. Similar material deposition, where the filler and substrate share compatible physical and chemical properties, generally aims for structural homogeneity. In contrast, dissimilar material deposition, such as the repair of SAE 1045 steel with 1Cr13 martensitic stainless steel, introduces complex metallurgical challenges. These include controlling the interfacial dilution rate—which determines the final chemistry of the fusion zone—and managing the thermal expansion mismatch between the base metal and the coating [4]. Consequently, scholars both domestically and internationally have maintained a consistent focus on this field in recent years, resulting in a continuous proliferation of research findings [5,6,7].

Furthermore, Fan successfully fabricated AlCoCrFeNi high-entropy alloy coatings on Q235 steel substrates. This study utilized an ultrasonic-assisted TIG cladding process to produce a denser coating microstructure and superior corrosion resistance, which significantly enhanced the surface properties of the substrate [8]. Similarly, Gao applied arc cladding technology to deposit Inconel 718 nickel-based superalloy coatings on Q345 carbon steel, imparting excellent wear and corrosion resistance to the material [9].

Due to the high versatility of WAAM materials, they have been implemented across a wide range of industrial sectors. The technology is no longer restricted to forming simple thin-walled components but is evolving toward the fabrication of parts with more intricate geometries and larger scales, seeing extensive application in aerospace, shipbuilding, mold repair, and other fields [10]. For instance, the National Aeronautics and Space Administration (NASA) innovatively developed an arc-based additive manufacturing process, which was successfully applied to the fabrication of liquid rocket engine liners and shrouds. This technology overcomes the bottlenecks associated with traditional casting and forging processes regarding complex structure formation, material properties, and manufacturing cycles, thereby attaining design and performance objectives that were previously unattainable [10]. Their research demonstrates that WAAM already possesses the capability to manufacture large-scale and complex structural components [10].

Taşdemir recognized the potential of WAAM in shipbuilding. He analyzed that both the materials used in WAAM and the process itself exhibit good adaptability. Leveraging its characteristics of low cost and fast forming, WAAM can effectively serve in manufacturing complex key components such as spherical parts and ship propellers, opening new directions for WAAM development [10,11].

Compared with cladding materials such as high-entropy alloys and nickel-based alloys, the chemical composition of iron-based alloy welding wire is closer to that of the SAE 1045 substrate, which results in a higher bonding strength between them. This not only achieves a better dilution rate but also forms a dense and stable metallurgical bond, endowing the cladding layer with higher bonding strength and comprehensive performance [12]. Due to the low cost of iron-based alloy welding wires, research on arc-clad iron-based alloy coatings is of greater practical engineering significance. 1Cr13 is a typical martensitic stainless steel that combines good toughness with high hardness and wear resistance. Its high chromium content provides excellent corrosion resistance, making it a durable and classic material [13]. In this study, a 1Cr13 stainless steel cladding layer was prepared on an SAE 1045 substrate using arc cladding technology. First, the hardness gradient from the surface to the substrate was measured to evaluate the mechanical transition. Subsequently, the friction coefficient curves and wear loss of both the cladding layer and the substrate were comparatively analyzed under identical conditions to quantify the improvement in wear resistance. Finally, the strengthening and wear mechanisms were investigated through metallographic observations and XRD phase analysis. This integrated approach provides a clearer understanding of the relationship between microstructure and tribological performance, offering a practical reference for the application of arc-clad iron-based coatings.

Despite the widespread use of arc processes, several knowledge gaps remain regarding the in situ repair of SAE 1045 steel with 1Cr13 coatings: (1) the microstructural evolution under the discrete thermal cycling of portable equipment is not fully understood; (2) the correlation between non-equilibrium solidification and millimeter-scale reinforcement integrity requires clarification; and (3) the load-dependent interfacial stabilization mechanisms under dry sliding conditions remain unexplored.

## 2. Materials and Methods

**Figure 1:**
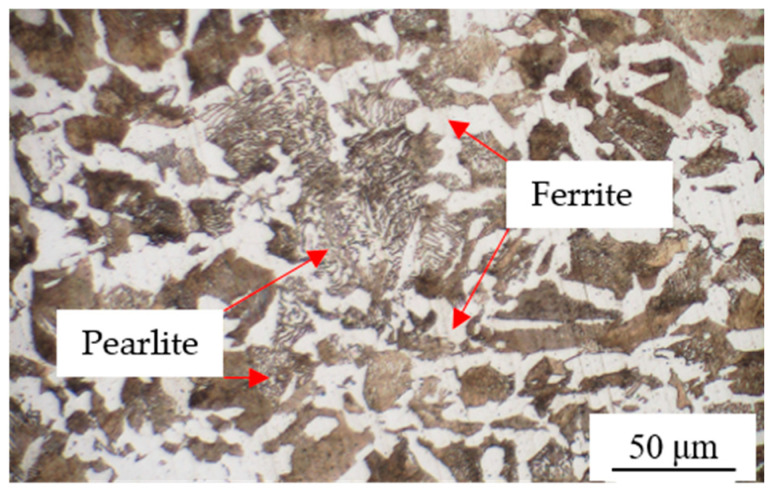
Microstructure of SAE 1045 substrate.

The nominal chemical compositions of the SAE 1045 substrate and the 1Cr13 filler wire are provided by the respective material suppliers in their quality certification reports. The substrate selected for the experiment was an SAE 1045 plate with dimensions of 100 mm × 100 mm × 10 mm. Its chemical composition is presented in Table 1. Prior to arc cladding, the surface was ground with sandpaper to remove rust, cleaned with acetone to eliminate oil stains, rinsed with ethanol, and dried with cool air. The microstructure morphology of the SAE 1045 substrate, revealed by etching with a 4% nital solution, is illustrated in Figure 1. It consists of white blocky ferrite and gray lamellar pearlite, with an average grain size of approximately 50 μm. The chemical composition of the 1Cr13 filler wire used in this study is listed in Table 2, and the wire diameter was 1.2 mm.

The experiment utilized an ESAB TIG 4300iw (Aristo TIG 4300iw, ESAB, Gothenburg, Sweden) argon arc welder as the heat source for arc cladding, equipped with a WE-007A automatic wire feeder (WE-007A automatic wire feeder, Wenzhou Suoli Welding Equipment Co., Ltd., Wenzhou, China) for synchronous wire feeding. Preliminary cladding trials were conducted to determine the range of process parameters. Using the response surface methodology (RSM), 17 sets of experiments were designed with cladding current, wire feed speed, and cladding speed as the primary parameters. In the optimization process, cladding width and dilution rate were selected as the response variables, with target values of 8 mm and 30%, respectively. These target values were determined based on preliminary single-factor experiments and practical forming quality requirements: a smaller dilution rate (≤30%) minimizes substrate dilution, thereby better preserving the properties of the filler wire, while a larger cladding width (≥8 mm) promotes a flatter bead surface, which facilitates subsequent multi-layer overlapping cladding. The analysis indicated that the cladding layer exhibited the best formability and comprehensive performance at a welding current of 220 A, a wire feed speed of 185 cm/min, and a cladding speed of 4 mm/s. High-purity argon was used to protect the molten pool, with the gas flow rate set at 12 L/min.

After the single-track arc cladding experiments, the specimens were cross-sectioned along the transverse direction using the wire electrical discharge machining (WEDM) method. The resulting specimens were ground, polished, and etched. The macroscopic morphology and microstructure of the cross-sections were observed and analyzed using a Hirox MXB-10RH-2000 3D digital microscope (MXB-10RH-2000 3D digital microscope, Hirox Co., Ltd., Tokyo, Japan). The surface hardness was measured with an HVS-1000B digital microhardness tester (HVS-1000B digital microhardness tester, Laizhou Huayin Testing Instrument Co., Ltd., Laizhou, China). The indentation interval was set to 150 μm, with a load of 4.9 N and a dwell time of 15 s. Each indentation was measured multiple times, and the arithmetic mean was recorded as the final result.

Multi-track overlap cladding experiments were performed using the optimized parameters (220 V current, 185 cm/min wire feed speed, and 4 mm/s cladding speed) with a 60% overlap rate. Following the experiments, the specimens were cut into 15 mm × 15 mm blocks along the longitudinal and transverse cross-sections. After the specimen surfaces were ground smooth with sandpaper and cleaned, phase analysis was conducted using a HD XpretPRO X-ray diffractometer (X’Pert PRO X-ray diffractometer, PANalytical B.V., Almelo, The Netherlands). Friction and wear performance of the cladding coating and the SAE 1045 substrate were tested using an MDW-02 reciprocating friction (MDW-02 reciprocating friction and wear tester, Jinan Yihua Tribology Testing Technology Co., Ltd., Jinan, China) and wear tester. Si3N4 ceramic balls with a diameter of 6.5 mm were used as the grinding media. The reciprocating stroke was set to 8 mm at a frequency of 2 Hz. To observe the stability of the friction coefficient, the wear duration was set to 30 min. Prior to testing, the specimen surfaces were ground smooth (Ra ≈ 0.08–0.12 μm) using SiC abrasive papers up to 3000 grit, rinsed with ethanol, and dried.

During the testing process, the friction coefficient–time curves were collected in real time via computer, and wear debris was collected using weighing paper after each test. Upon completion, the morphology and chemical composition of the worn surfaces and debris were analyzed using a SIGMA 500 scanning electron microscope (SIGMA 500 scanning electron microscope, Carl Zeiss AG, Jena, Germany) equipped with an energy-dispersive spectrometer (EDS). Before the wear tests, specimens were ultrasonically cleaned in ethanol, dried, and weighed. After the tests, the specimens were again ultrasonically cleaned to remove residual debris and weighed to calculate the wear weight loss.

## 3. Results

### 3.1. Microstructure, Phase Composition, and Hardness of the Arc Cladding Layer

#### 3.1.1. Microstructural Characteristics of the Arc Cladding Layer

**Figure 2:**
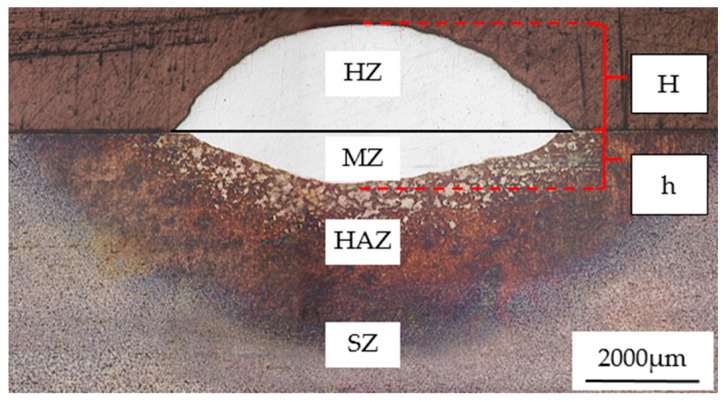
Macroscopic morphology of the cladding layer cross-section.

Figure 2 shows the macroscopic morphology of the cladding layer cross-section. As shown in the figure, the cladding layer is divided into four distinct regions from top to bottom: cladding zone (CZ), melting zone (MZ), heat-affected zone (HAZ), and substrate zone (SZ); the cladding layer thickness (H) is approximately 1.8 mm, and the penetration depth (h) is approximately 0.66 mm.

**Figure 3:**
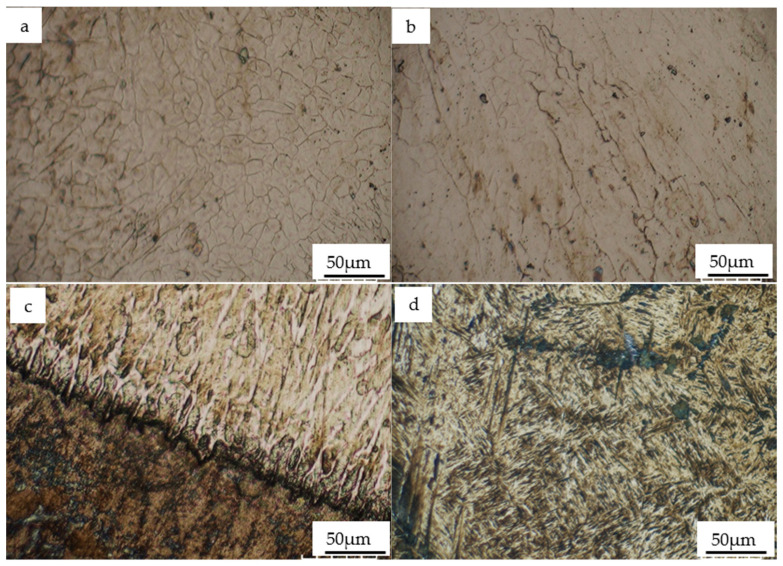
Microstructure of the arc cladding layer: (**a**) top; (**b**) middle; (**c**) bottom; and (**d**) heat-affected zone (HAZ).

The metallographic structure of the cladding layer, revealed by etching with aqua regia, is shown in Figure 3. Images (a) to (d) illustrate the microstructural evolution from the cladding zone to the heat-affected zone (HAZ). Influenced by the cooling rate, the morphology evolves from columnar crystals at the bottom to dense equiaxed crystals at the top, with an average grain size of less than 20 μm. According to solidification theory, the constitutional supercooling during crystal growth is the decisive factor for crystal morphology, which is determined by the ratio of the temperature gradient *G* to the solidification rate R [14,15]. During the cladding process, the high-temperature molten pool spreads rapidly across the substrate. Contact with the cold substrate induces a quenching effect, creating a massive temperature difference and an immediate high temperature gradient. Due to the high temperature on the liquid side and the slow growth rate, the G/R value at the bottom of the molten pool is very large, resulting in a solidification structure primarily composed of cellular and columnar crystals [15].

As the solid/liquid interface moves from the bottom toward the center of the molten pool during solidification, it encounters newly solidified metal at higher temperatures, causing the temperature gradient G to decrease gradually. Simultaneously, the molten pool itself continues to cool, leading to a corresponding increase in the solidification rate *R*. This results in a continuous decrease in the G/R ratio, which reduces the relative grain size and drives the evolution from cellular crystals to dendrites [14,15]. Further observation of Figure 3b,c reveals distinct directional growth characteristics in the bottom and middle zones of the molten pool. This orientation occurs because the primary heat dissipation channels in these areas are the solidified metal and the metal substrate. Crystal growth is fastest in the direction perpendicular to the interface at the bottom of the molten pool, resulting in significant directional alignment [15]; the average dendritic spacing of the columnar dendrites is recorded at less than 30 µm.

Figure 3a displays the top of the cladding layer, where the microstructure consists mainly of fine, dense equiaxed crystals with no obvious growth direction. This is primarily due to two factors: first, heat dissipation at the top relies on both the air and the solidified metal; second, growth is disturbed by melt convection within the molten pool. In this top region, the temperature gradient reaches its minimum while the solidification rate rises to its maximum, resulting in the smallest G/R ratio. The high constitutional supercooling in this zone promotes the formation and growth of a large number of crystal nuclei within the melt, creating the necessary conditions for the emergence of fine equiaxed crystals [14].

The microstructure of the substrate heat-affected zone (HAZ) below the cladding layer is shown in Figure 3d, where a typical lath martensite structure can be clearly observed. During the cladding process, due to its proximity to the high-temperature molten pool, the austenite within this region begins to transform even though the temperature is insufficient to melt the material. After transforming into austenite at high temperatures, the material undergoes a phase transformation into high-hardness lath martensite. This is primarily attributed to the inherent high hardenability of the 1Cr13 martensitic stainless steel, which facilitates air-cooling self-quenching during the cooling process. While the thermal conduction of the bulk metal substrate contributes to the cooling rate, the material’s intrinsic capability for martensitic transformation is the decisive factor [16].

#### 3.1.2. Phase Analysis of the Arc Cladding Layer

**Figure 4:**
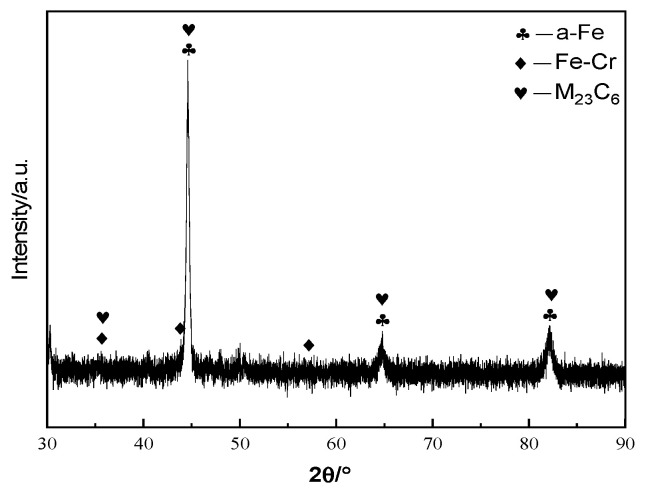
X-ray diffraction (XRD) pattern of the arc cladding layer.

Figure 4 shows the X-ray diffraction (XRD) pattern of the arc cladding layer. It can be observed that the cladding layer primarily consists of α-Fe martensite, an Fe-Cr solid solution, and a small amount of M_23_C_6_ (M = Cr, Fe) carbides. Due to the high Cr content (up to 13%) in the cladding wire, the cladding layer exhibits good hardenability, which facilitates the predominant transformation of austenite into martensite during the arc cladding process. Additionally, because the cooling rate of the molten pool is extremely high, the solid solubility of the alloying element Cr is significantly improved. Consequently, the phase composition of the cladding layer is mainly composed of α-Fe martensite and an Fe-Cr solid solution [16].

#### 3.1.3. Hardness Analysis

**Figure 5:**
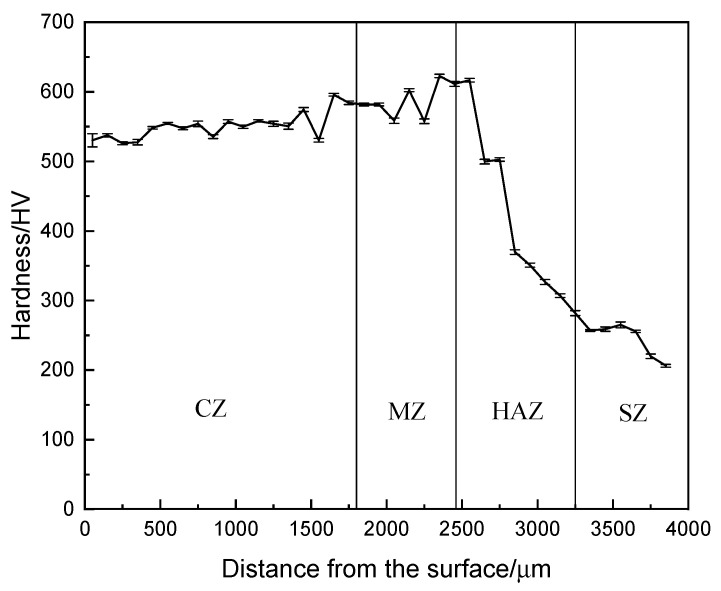
Hardness profile of the arc cladding layer.

As shown in Figure 5, the microhardness distribution curve from the surface to the interior of the arc cladding layer indicates that the average hardness of the outermost cladding zone (CZ) reaches 551.94 HV0.2. This value significantly exceeds the substrate hardness (243.97 HV0.2), being approximately 2.26 times that of the substrate. According to Figure 3a,b, the microstructure of the CZ is uniform and dense, with the average grain size of the equiaxed crystals in the upper region being less than 20 μm and the average dendritic spacing of the columnar dendrites in the middle being less than 30 μm. It is evident that the grain size of the cladding layer is significantly refined compared to the SAE 1045 substrate. The XRD pattern in Figure 4 further reveals that the cladding layer is composed of high-hardness α-Fe martensite, an Fe-Cr solid solution, and a small amount of M23C6 carbides. The significant enhancement in strength and hardness is attributed to the combined contributions of fine-grain strengthening, martensitic transformation strengthening, solid solution strengthening, and carbide precipitation strengthening [16,17].

Interestingly, the peak hardness was observed in the melting zone (MZ) rather than the cladding zone (CZ). This is primarily attributed to the diffusion of carbon from the SAE 1045 substrate into the melt pool, which increases the carbon supersaturation of the martensite in the MZ. Furthermore, the high cooling rate at the fusion boundary promotes grain refinement. The synergistic effect of carbon enrichment and fine-grained strengthening results in the MZ’s hardness exceeding that of the alloy-rich CZ.

While previous studies on carbon steels emphasize that higher carbon (C) content directly increases martensite hardness [13], this relationship must be re-evaluated for high-alloy steels. In this study, although the C content of the 1Cr13 wire is lower than that of the SAE 1045 substrate, the high chromium (Cr) content in the 1Cr13 coating significantly increases the carbon equivalent and the hardenability of the alloy. This enables the formation of a fully martensitic structure with high hardness (551.94 HV0.2), which far exceeds the hardness of the ferrite–pearlite matrix in the substrate (243.97 HV0.2). The dilution effect of the SAE 1045 substrate significantly affects the local carbon concentration across the interface. During arc cladding, the partial melting of the substrate (0.45% C) promotes the diffusion of carbon into the molten pool, while the chromium content from the 1Cr13 coating is slightly diluted. This elemental redistribution creates a narrow transition zone, which is consistent with the observed microhardness gradient near the fusion line. In the CZ, the closer the region is to the melting zone (MZ), the higher the hardness of the generated martensite. Consequently, as shown in Figure 5, the hardness curve of the CZ is relatively lower in the initial portion and exhibits an upward trend toward the rear [13]. Further observation of Figure 5 shows that the hardness of the MZ is slightly higher than that of the CZ, primarily because the higher C content from the substrate leads to the formation of harder martensite [13]. Additionally, the hardness curve of the heat-affected zone (HAZ) shows obvious fluctuations. The upper part of the HAZ exhibits higher hardness due to the quenching phenomenon, resulting in a lath martensite structure with high hardness, as illustrated in Figure 3d. As the distance from the bottom of the molten pool increases, the temperature in the HAZ gradually decreases, leading to a lower transformation rate of austenite. Upon cooling, the martensite content within the internal structure decreases, and the hardness value gradually drops to that of the substrate [16,18].

### 3.2. Friction and Wear Performance of the Arc Cladding Layer

#### 3.2.1. Friction Coefficient

**Figure 6:**
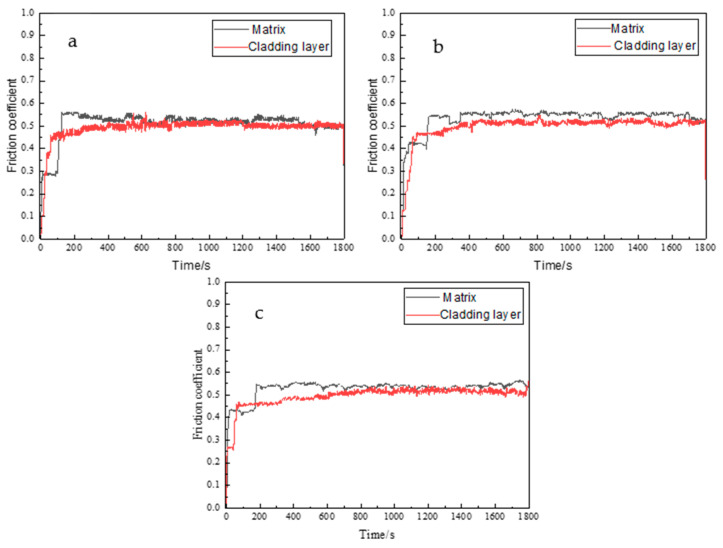
Friction coefficient curves of the substrate and the cladding layer: (**a**) 10 N; (**b**) 50 N; and (**c**) 100 N.

To evaluate the friction and wear performance of the arc cladding layer, comparative experiments were conducted between the arc cladding layer and the SAE 1045 substrate. Data on the variation in the friction coefficient over time were collected in real time during the wear tests. Figure 6 illustrates the friction coefficient curves as a function of time for the substrate and the cladding layer under loads of 10, 50, and 100 N. Observation of the curves reveals that both materials exhibit a running-in stage and a stable wear stage. The rapid increase in the friction coefficient during the running-in stage is attributed to the small initial contact area and high contact stress between the grinding ball and the surface. As the experiment progresses, the contact area increases, and the curves gradually stabilize, marking the entry into the stable wear stage [19].

As shown in Figure 6, at the beginning of the running-in stage, the friction coefficient curve of the substrate shows a distinct transient stage of very short duration, followed by a rapid rise to a stable value. This transient behavior is caused by the accumulation of debris [20]. Based on the time axis, the cladding layer enters the stable wear stage at approximately 100 s. Between 100 s and 600 s, the friction coefficient increases slowly until reaching a relatively stable value. In contrast, the substrate enters the stable wear stage between 140 s and 200 s, after which its friction coefficient directly reaches a stable value. This difference occurs because the hardness of the substrate is significantly lower than that of the cladding layer. Under the same load and duration, the wear on the substrate surface is much more severe, requiring a longer transition period to reach the stable wear stage [21]. Since the cladding layer possesses higher hardness and a friction-reducing effect, the friction coefficient exhibits a slow increasing process during the stable wear stage as wear debris gradually accumulates.

As seen in Figure 6a–c, the friction coefficient curves stabilize after 200 s. Therefore, the data after 200 s were used to calculate the average values. Under loads of 10, 50, and 100 N, the average friction coefficients of the substrate during the stable wear stage were 0.532 ± 0.018, 0.548 ± 0.031, and 0.538 ± 0.046, respectively. These values are somewhat higher than the average friction coefficients of the arc cladding layer, which were 0.502 ± 0.007, 0.511 ± 0.012, and 0.506 ± 0.01. This confirms that the arc cladding layer provides a certain friction-reducing effect.

To ensure the reliability and reproducibility of the tribological data, friction and wear tests under each load (10 N, 50 N, and 100 N) were performed in triplicate. The average friction coefficient (COF) and its corresponding standard deviation (SD) were calculated from the last 500 data points of the steady-state wear stage (see Table 3). As summarized in Table 3, the 1Cr13 cladding layer exhibits a significantly narrower SD range (0.007–0.039) compared to the SAE 1045 substrate (0.018–0.072) across all testing conditions, reflecting a more stable and consistent friction interface provided by the hardened martensitic matrix.

#### 3.2.2. Wear Weight Loss

**Figure 7:**
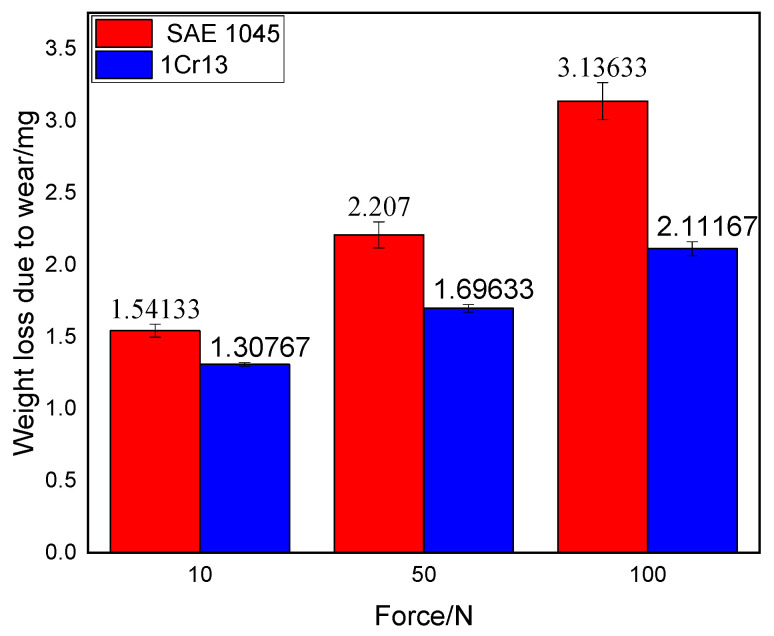
Wear weight loss of the substrate and 1Cr13 cladding layer under different loads.

Prior to the wear test, the specimens were immersed in ethanol for ultrasonic cleaning, dried, and then weighed. Following the wear test, the same ultrasonic cleaning procedure was performed to remove residual wear debris from the surface, followed by a second weighing. To minimize experimental error, each sample was measured multiple times, and the arithmetic mean was calculated as the final result. Figure 7 presents the calculated results for the wear weight loss. Under different applied loads, the average wear weight losses for the substrate were 1.589 mg, 2.153 mg, and 3 mg, respectively, while those for the cladding specimens were 1.32 mg, 1.726 mg, and 2.133 mg. The wear loss of the cladding layer accounted for 83.07%, 80.16%, and 71.1% of that of the substrate, respectively. These results indicate that the wear resistance of SAE 1045 is significantly improved after the arc cladding of the 1Cr13 coating.

Although the 1Cr13 cladding layer exhibits a remarkable 2.26-fold increase in microhardness compared to the SAE 1045 substrate, its volumetric wear resistance improves by a more moderate 17–29%. This non-linear relationship suggests that hardness is not the sole determinant of wear performance in this system. The high-hardness martensite, while resisting initial abrasive plowing, may undergo micro-cracking or delamination due to its limited toughness under intense repetitive loading.

To provide a more comprehensive comparison, the specific wear rate (ω) was calculated by normalizing the wear volume against the applied load and total sliding distance using the formula

(1) $$\omega =\frac{V}{F\times L}$$

*ω* is the specific wear rate (mm^3^/N·m), V is the wear volume loss (mm^3^), *F* is the applied normal load(*N*), and L is the total sliding distance(m).

Under loads of 10, 50, and 100 N, the specific wear rates of the 1Cr13 cladding layer were 14.78 × 10^−5^ mm^3^/N·m, 3.78 × 10^−5^ mm^3^/N·m, and 2.39 × 10^−5^ mm^3^/N·m, respectively. These values were consistently lower than those of the SAE 1045 substrate (17.57 × 10^−5^ mm^3^/N·m, 4.76 × 10^−5^ mm^3^/N·m, and 3.32 × 10^−5^ mm^3^/N·m). Notably, the specific wear rate for both materials decreased with increasing load, suggesting that the resistance to unit load improves under higher-pressure conditions, which is consistent with the enhanced stability of the friction pair at higher loads.

#### 3.2.3. Wear Mechanism Analysis

**Figure 8:**
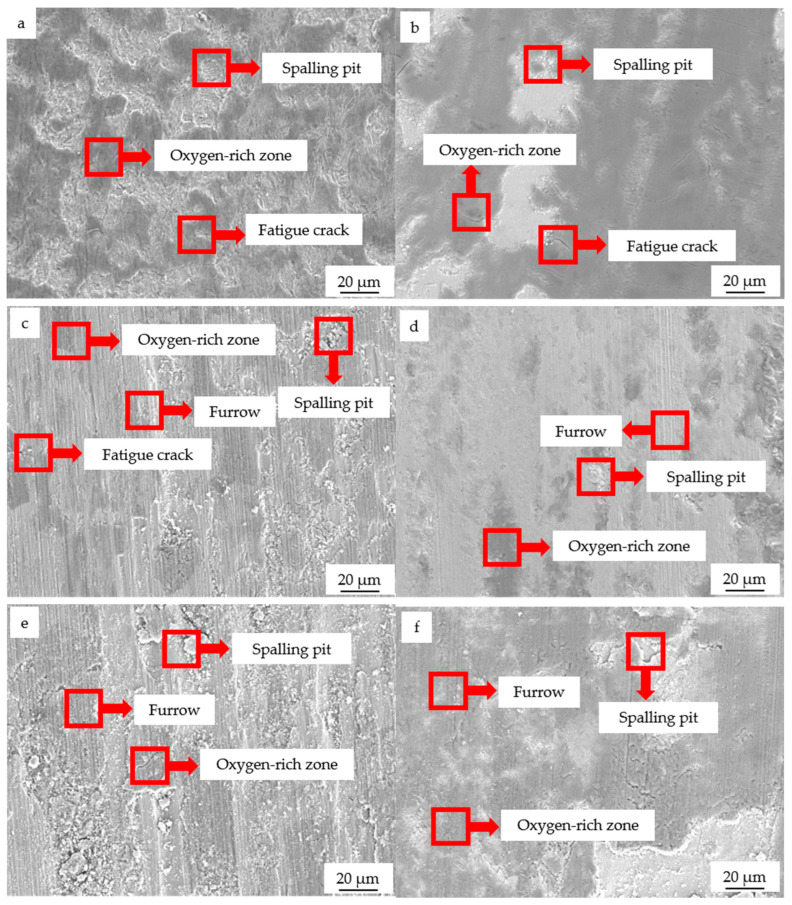
Wear morphology of the substrate and the cladding layer under different loads. Substrate: (**a**) 10 N, (**c**) 50 N, and (**e**) 100 N; cladding layer: (**b**) 10 N, (**d**) 50 N, and (**f**) 100 N.

Figure 8 presents the SEM morphologies of the worn surfaces for both the SAE 1045 substrate and the arc cladding layer under loads of 10, 50, and 100 N. It is evident from Figure 8 that prominent furrows appear on both surfaces at loads of 50 and 100 N, indicating the presence of abrasive wear. The formation of these furrows results from the asperities on the grinding ball and the wear debris generated during friction against the counterface. Upon oxidation, these particles form high-hardness oxides that are pressed into the counterface under normal loads. During continuous reciprocating friction, they shear and cut the specimen surface, leading to the formation of groove-like wear marks [22,23]. Compared to the SAE 1045 surface, the furrows on the worn surface of the arc cladding layer are significantly finer and shallower [16]. This is primarily attributed to the higher hardness and strength of the cladding layer, which prevents the asperities of the grinding ball and debris particles from easily pressing into the surface, thereby mitigating the degree of plastic plowing [24,25]. This further validates that the arc cladding layer possesses superior wear resistance compared to the SAE 1045 substrate.

As shown in Figure 8, delamination pits are observed on the wear marks of both materials, suggesting the presence of an adhesive wear mechanism. Comparative analysis reveals that, under various loads, the delamination pits on the SAE 1045 substrate surface are larger in area and more densely distributed, whereas those on the cladding layer are smaller and more sparse. During the friction and wear process, adhesion points form between the grinding ball and the substrate surface. Since the hardness of the SAE 1045 substrate is far lower than that of the grinding ball, the substrate material at the adhesion points is sheared and peeled off during subsequent relative sliding, resulting in delamination pits. In contrast, the hard phases within the cladding layer effectively suppress local plastic deformation, thereby preventing the formation of large-scale delamination pits [22,25]. Further observation of Figure 8 indicates the presence of dark, oxygen-enriched zones on the wear marks of both the SAE 1045 substrate and the cladding layer, demonstrating that oxidative wear occurred in both materials during the wear process [24].

**Figure 9:**
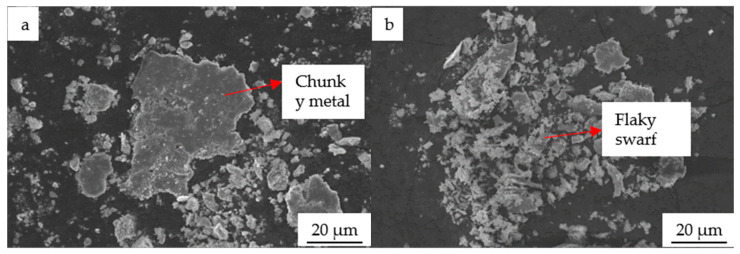
Morphology of wear debris for the substrate and the cladding layer: (**a**) wear debris of the substrate; (**b**) wear debris of the cladding layer.

The morphology of wear debris under a load of 100 N is the most representative. Figure 9a,b illustrate the morphology of wear debris for the SAE 1045 and the cladding layer under a load of 100 N, respectively. As shown in the figures, most of the wear debris appears in blocky or flocculent forms. Comparing the sizes of the wear debris, it is evident that the debris from the cladding layer is relatively smaller. According to Bhushan’s tribology theory, smaller debris size corresponds to lower wear, which further demonstrates the superior wear resistance of the cladding layer [26].

**Figure 10:**
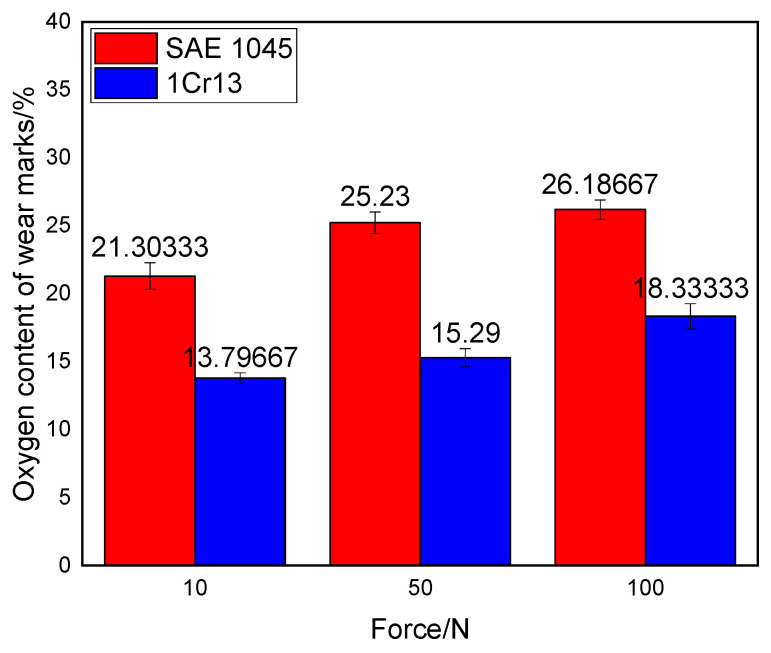
Oxygen content bar chart of the wear marks of the substrate and the cladding layer.

During the wear experiments, the observation of distinct brownish-tan wear debris confirms that an oxidative wear mechanism occurred in both the SAE 1045 substrate and the cladding layer. The elemental composition of the worn surfaces was detected using SEM, and the results are presented in Figure 10. It can be observed that both the SAE 1045 substrate and the cladding layer surfaces underwent varying degrees of oxidation. Based on the EDS analysis results, the oxygen content in the worn regions of the SAE 1045 substrate under loads of 10, 50, and 100 N was 20.5%, 25.04%, and 26.38%, respectively. In contrast, the oxygen content at the wear marks of the cladding layer was 14.2%, 15.4%, and 17.35%. The data clearly indicates that the cladding layer maintained a lower degree of oxidation during the wear process under different loads.

During the friction and wear process, high temperatures are generated on the friction pair surfaces due to intense friction, leading to a reaction with oxygen in the air and subsequent oxidative wear. The arc cladding layer contains a high concentration of Cr, which reacts with oxygen to form a dense Cr_2_O_3_ layer on the surface. This layer effectively inhibits the bonding of the friction surface with oxygen [27,28], and consequently, compared to the SAE 1045, the oxygen content on the worn surface of the cladding layer is significantly reduced.

The quantitative EDS results (Table 4) illustrate a load-dependent material transfer mechanism. The 1Cr13 cladding layer consistently exhibited higher Si concentrations than the substrate, particularly at 100 N where the Si content reached 5.25 wt.% (3.55 times that of the substrate). This is primarily attributed to the high shear strength and hardness of the martensitic structure in the coating, which accelerates the micro-fragmentation of the Si_3_N_4_ ball. The detached ceramic particles are subsequently embedded into the wear track, acting as a protective hard-phase layer. This synergistic interaction between the high-hardness coating and the ceramic counterbody is a key characteristic of the 1Cr13 cladding layer’s superior tribological performance.

## 4. Discussion

In this study, a high-performance 1Cr13 coating was successfully prepared on SAE 1045 steel via arc cladding, fulfilling the research objective of developing an efficient surface reinforcement method for mechanical components. The microhardness (551.94 HV0.2) and wear resistance are significantly enhanced due to the high hardenability of the 1Cr13 alloy. Compared to previous laser cladding methods [16], this arc cladding approach demonstrates unique advantages in heavy-load industrial repairs by providing a robust metallurgical bond and a larger remelting area.

### 4.1. Strengthening Mechanisms and Hardness Enhancement

The microhardness of the 1Cr13 cladding layer (551.94 HV0.2) is significantly higher than that of the SAE 1045 substrate (243.97 HV0.2). This substantial hardening effect is primarily attributed to the formation of a dense lath martensite structure and the high carbon supersaturation within the lattice. Furthermore, the rapid solidification during the TIG cladding process leads to grain refinement, which, according to the Hall–Petch relationship, further enhances the resistance to plastic deformation compared to the coarse ferrite–pearlite microstructure of the substrate. While the localized thermal cycling during multi-pass arc cladding may induce the precipitation of fine M_23_C_6_ carbides, contributing to a degree of dispersion strengthening, the dominant hardening mechanism in this study is primarily attributed to martensitic lattice strengthening and Cr/C solid solution strengthening. This is evidenced by the sharp α-Fe diffraction peaks and the characteristic dense lath morphology observed in our results.

### 4.2. Wear Resistance and Mechanism Analysis

The friction and wear tests indicate that the cladding layer exhibits improved wear resistance compared to the substrate under the tested loads (10 N, 50 N, and 100 N). The lower average friction coefficients (0.502–0.511) and the reduced wear weight loss (accounting for 71.1% to 83.07% of the substrate’s loss) reflect a stable tribological interaction. Furthermore, the standard deviation (SD) values calculated from the steady-state stage (Table 3) provide additional evidence for the data’s reliability. The narrower SD ranges of the cladding layer (0.007–0.039) in comparison with the substrate (0.018–0.072) demonstrate a reduction in signal fluctuations, suggesting a consistent friction behavior throughout the test.

The wear mechanism analysis (Figure 8) provides deeper insights into these performance gains:

- Abrasive Wear: The higher hardness of the cladding layer prevents the asperities of the grinding ball and hard oxide debris from pressing deeply into the surface, resulting in narrower and shallower furrows compared to the substrate.
- Adhesive Wear: While both materials exhibited delamination pits, the cladding layer effectively inhibited large-scale plastic deformation due to its high yield strength, leading to smaller and more dispersed pits.
- Oxidative Wear: The reduced oxygen content (14.2–17.35%) in the cladding layer’s wear marks suggests that the high Cr content plays a pivotal role. The formation of a dense Cr_2_O_3_ protective film during the high-temperature friction process effectively isolates the metal from the air, mitigating oxidative degradation.

In summary, the 1Cr13 arc cladding layer provides a multi-layer defense—high hardness to resist abrasive cutting, high strength to resist adhesive delamination, and a chemical barrier (Cr-oxide) to resist oxidative wear—thereby significantly extending the potential service life of SAE 1045 components in abrasive and oxidative environments. It is noteworthy that the 2.26-fold increase in hardness resulted in a relatively moderate improvement in wear resistance (17–29%). This discrepancy is attributed to the inherent brittleness of the untempered martensite in the as-cladded state. While the high hardness resists micro-cutting, the limited fracture toughness of fresh martensite may lead to localized micro-fragmentation under high contact loads, which partially offsets the benefits of the hardness increment.

### 4.3. Analysis of Mechanical Integrity and Potential Failure Mechanisms

The long-term mechanical integrity of the 1Cr13 cladding layer is governed by the interplay between its high hardness and martensitic brittleness. Under thermal cycling, the primary failure mode is expected to be thermal fatigue cracking initiated at microstructural inhomogeneities. However, the continuous metallurgical transition observed in this study minimizes the stress concentration at the fusion line. Furthermore, to mitigate brittle spalling, a post-cladding tempering heat treatment could be employed in practical applications to transform the fresh martensite into tempered martensite. Regarding mechanical integrity, the larger remelting zone and slower cooling rate of the TIG process (compared to laser cladding) facilitate a more gradual thermal gradient. This characteristic helps in redistributing residual tensile stresses and ensuring a more robust metallurgical bond, which is critical for preventing delamination during long-term service in heavy-load industrial repairs, thereby optimizing the balance between wear resistance and fracture toughness without significantly compromising the hard-phase benefits.

## 5. Conclusions

In this study, a 1Cr13 cladding layer was fabricated on an SAE 1045 substrate via the arc cladding process. The microstructure, phase composition, microhardness, and friction and wear performance of the cladding layer were systematically investigated. The main conclusions are as follows.

The arc cladding layer is primarily composed of alpha-Fe martensite, an Fe-Cr solid solution, and a small amount of M_23_C_6_ carbides. The microstructure evolves from columnar crystals at the bottom to fine equiaxed crystals at the top (average grain size < 20 μm) due to the variation in the temperature gradient (G) and solidification rate (R).

The cladding zone (CZ) exhibits a refined microstructure dominated by dense lath martensite, resulting in an average microhardness of 551.94 HV0.2. This represents a 2.26-fold increase relative to the SAE 1045 substrate (243.97 HV0.2). The enhancement is quantitatively consistent with the combined effects of martensitic transformation strengthening—driven by the high carbon equivalent and hardenability of the 1Cr13 alloy—and solid solution strengthening from the high Cr content. Furthermore, the rapid thermal cycle of the arc process facilitates fine-grain strengthening, which collectively provides a robust structural basis for the observed improvement in surface mechanical properties.

The cladding layer exhibits improved friction and wear performance compared to the substrate. Under loads of 10, 50, and 100 N, the average friction coefficients of the cladding layer (0.502–0.511) are consistently lower than those of the substrate (0.532–0.548). The wear weight loss of the cladding layer is approximately 71.1–83.07% of the substrate’s loss, demonstrating an enhancement in wear resistance.

The wear mechanisms for both the substrate and the cladding layer involve a combination of abrasive wear, adhesive wear, and oxidative wear. However, the higher hardness of the cladding layer effectively mitigates plastic plowing and delamination. Furthermore, the high Cr content promotes the formation of a dense Cr_2_O_3_ protective film, which significantly reduces the oxygen content on the worn surface (14.2–17.35%) compared to the substrate (20.5–26.38%), thereby inhibiting oxidative wear.

It should be noted that the present investigation is confined to dry sliding at room temperature. In practical applications involving high temperatures or lubricated contacts, the performance of the 1Cr13 cladding layer may be further influenced by thermal softening or the stability of the lubricant film. Future research will focus on the tribological evolution of these coatings under extreme environments to fully explore their industrial potential.

## Figures and Tables

**Table 1:** Chemical composition of SAE 1045 substrate (wt. %).

C	Si	Cr	Ni	Mn	P	S	Fe
0.46	0.27	0.05	0.04	0.59	0.024	0.016	Bal

**Table 2:** Chemical composition of 1Cr13 welding wire (wt. %).

C	Si	Cr	Ni	Mn	P	S	Fe
0.15	0.41	13.05	0.26	0.29	0.023	0.011	Bal

**Table 3:** Range of the standard deviation of the friction coefficient in the stable phase.

	10 N	50 N	100 N
SAE 1045	0.018–0.034	0.031–0.052	0.046–0.072
1Cr13	0.007~0.014	0.012~0.028	0.021~0.039

**Table 4:** Comparative analysis of Si element concentration (wt.%) on the wear tracks.

Load (N)	Sample Type	Point 1	Point 2	Point 3	Average
10	Substrate	4.44	5.08	6.31	5.28
	Cladding layer	16.96	12.78	11.25	13.66
50	Substrate	1.53	1.21	1.60	1.45
	Cladding layer	2.40	1.93	2.04	2.12
100	Substrate	1.56	1.43	1.55	1.48
	Cladding layer	5.38	5.16	5.22	5.25

## Data Availability

The original contributions presented in this study are included in the article. Further inquiries can be directed to the corresponding author.
